# Expression and localization of SpoIISA toxin during the life cycle of *Bacillus subtilis*

**DOI:** 10.1016/j.resmic.2010.09.005

**Published:** 2010-11

**Authors:** Stanislava Rešetárová, Patrik Florek, Katarína Muchová, Anthony J. Wilkinson, Imrich Barák

**Affiliations:** a Institute of Molecular Biology, Slovak Academy of Sciences Dúbravská cesta 21, 845 51 Bratislava, Slovakia; b Structural Biology Laboratory, Department of Chemistry, University of York, York YO10 5YW, UK

**Keywords:** Toxin–antitoxin systems, *SpoIIS* locus, *Bacillus subtilis*

## Abstract

The previously identified *spoIIS* locus encodes a toxin–antitoxin system in *Bacillus subtilis*. It comprises two genes, s*poIISA* encoding a toxin and s*poIISB* encoding an antitoxin, which lies adjacent to each other on the chromosome. Each of the *spoIIS* coding sequences is preceded by a promoter region and the two genes together constitute an operon. The function of SpoIISA is unknown, although it has been shown that the absence of SpoIISB or loss of its function leads to a block in sporulation at stage II. The cytoplasmic membrane has been proposed as the target of the SpoIISA toxin. Heterologously expressed SpoIISA–SpoIISB was shown to be functional in *Escherichia coli*, where again the cytoplasmic membrane was the most probable target for SpoIISA toxicity. Here we analyzed the effects of SpoIISA production during vegetative growth of *B. subtilis* and during sporulation by following the levels of SpoIISA. SpoIISA levels increase at the point of entry into stationary phase of cell cultures grown in sporulation-inducing medium. However, SpoIISA expression appears to be unrelated to the sporulation process, since it is independent of the major early sporulation-specific transcription factor, Spo0A.

We also investigated SpoIISA localization within the cell. We confirmed the predicted localization of SpoIISA at the *B. subtilis* cytoplasmic membrane. In addition, we observed localization of SpoIISA in higher level structures in a cell-wall-dependent manner.

## 1 Introduction

Programmed cell death (PCD) as a process of genetically regulated cell suicide has importance in eukaryotes, but there is growing evidence that PCD also plays a significant role in developmental processes in bacteria (Doyle and Koch, 1987; Foster, 1992; Kuroda et al., 1993; Rosenbluh and Rosenberg, 1989; Mueller and Dworkin, 1991). Some of the proteins which are thought to elicit PCD in bacteria belong to the family of addiction modules, also known as toxin–antitoxin (TA) systems. TA systems, which generally consist of stable toxin and labile antitoxin, were first discovered on low copy plasmids of *Escherichia coli*, where they are responsible for the phenomenon of post-segregational killing in plasmid-free cells (Engelberg-Kulka and Glaser, 1999). Subsequently, a large number of homologues of plasmid-encoded TA systems have been found on the chromosomes of bacteria (Masuda et al., 1993; Aizenman et al., 1996; Gotfredsen and Gerdes, 1998; Mittenhuber, 1999) and Archaea (Pandey and Gerdes, 2005). TA systems are generally divided into two groups that differ in the mechanism of toxin inactivation; in type I systems, the toxin is neutralized by inhibition of its translation, where small antisense RNA acts as an antitoxin; in type II systems, both the toxin and the antitoxin are proteins and the toxin is inactivated by binding to its antidote (Hayes, 2003).

*spoIIS* is a chromosomally encoded TA system in the Gram-positive bacterium *B. subtilis*. It is composed of two genes, *spoIISA* and *spoIISB*, constituting an operon which resembles type II TA systems. SpoIISA toxin consists of 248 amino acid residues; the first third of the protein contains three putative transmembrane segments and the protein is predicted to lie in the cytoplasmic membrane. The C-terminal two-thirds of the protein are predicted to be localized in the cytoplasm. The SpoIISB antitoxin, consisting of 56 amino acid residues, is a small cytosolic protein that neutralizes the lethal effect of SpoIISA by forming a SpoIISA–SpoIISB complex (Adler et al., 2001). Previous studies revealed that the cytoplasmic membrane could be the target of the toxic action of SpoIISA. The effect of SpoIISA overexpression in the absence of the SpoIISB antidote has been studied both in *B. subtilis* and *E. coli*. In *B. subtilis*, large plasmolysis zones and holes in the peptidoglycan layer were observed in cells with complete or incomplete septa, suggesting that the SpoIISA toxin could act as a holin (Adler et al., 2001). The disruption of the cytoplasmic membrane was also seen in *E. coli* cells (Florek et al., 2008). It was also shown that intact SpoIISA is integral to cell killing activity, since neither the cytosolic region nor the transmembrane domain alone can exert a toxic effect (Florek et al., 2008).

Previous genetic analysis identified mutations in the *spoIIS* locus that affect sporulation of *B. subtilis* (Adler et al., 2001). Sporulation in *B. subtilis* is a developmental process triggered by nutrient deprivation, which starts with asymmetric cell division and leads to formation of dormant spores (Piggot and Coote, 1976; Losick and Stragier, 1992). The process of spore formation is divided into seven principal stages associated with characteristic morphological changes: polar septum formation, engulfment of the forespore, formation of the cortex and spore coat, maturation of the spore and lysis of the mother cell (Errington, 2003). Mutations in SpoIISA or in SpoIISB as well as deletion of SpoIISB cause a block of sporulation after septum formation at stage II. However, deletion of *spoIISA* alone or deletion of the whole locus does not affect sporulation (Adler et al., 2001).

Adler and colleagues also identified two promoters in the *spoIIS* locus; the first drives transcription of both genes, while the second directs expression of *spoIISB* alone. Analysis of P*_AB_* and P*_B_* promoter activity using *lacZ* fusions revealed that the P*_AB_* promoter is weaker than the P*_B_* promoter and both are σ^A^-dependent promoters (Adler et al., 2001). However, subsequent analyses showed that expression of *spoIISA* may be transcribed by RNA polymerase containing later-acting sporulation sigma factor σ^K^, which controls expression of genes 3–4 h after the onset of sporulation (Steil et al., 2005).

The physiological role of SpoIISA and the conditions under which it is expressed are unknown. Here, using western blot analysis, we monitored the levels of SpoIISA in *B. subtilis* during its life cycle. Our results show that SpoIISA is present in cells during stationary phase of growth and during the early stages of sporulation. We measured SpoIISA levels in different growth media, revealing that the toxin is present in higher amounts when cells were grown in sporulation medium compared to LB medium. We confirmed the presence of SpoIISA in a *spo0A* mutant of *B. subtilis*, indicating that activation of expression of the toxin is independent of sporulation sigma factors. Using GFP fused to the transmembrane domain of SpoIISA, we demonstrate that the SpoIISA protein is localized in the cytoplasmic membrane.

## 2 Materials and methods

### 2.1 Bacterial strains, plasmids and culture conditions

*E. coli* strain MM294 (Backman et al., 1976), used for DNA manipulation, was cultured in Luria–Bertani (LB) medium (Ausubel et al., 1987) at 37 °C. Until otherwise indicated, *B. subtilis* strains were grown in LB or Difco sporulation medium (DSM; Schaeffer et al., 1965) at 37 °C. Ampicillin (100 μg mL^−1^) and chloramphenicol (40 μg mL^−1^) were used for plasmid selection in *E. coli*, and kanamycin (10 μg mL^−1^) and spectinomycin (100 μg mL^−1^) were used for selecting transformants harboring plasmids integrated into the *B. subtilis* chromosome. To induce expression of C-terminal fusion of the SpoIISA membrane part with GFP under control of the P*_xyl_* promoter, all media were supplemented with 0.5% (w/v) xylose. *B. subtilis* protoplasted cells were prepared by resuspension in 1×SMM buffer (0.5 M sucrose, 20 mM maleic acid, 20 mM MgCl_2_, pH 6.5) containing 4 mg ml^−1^ lysozyme as described previously (Errington, 1990).

All bacterial strains and plasmids used in this study are listed in Table 1.

### 2.2 Construction of recombinant plasmids and bacterial strains

The sequences of PCR primers used in this work are shown in Table 2.

Employing *B. subtilis* strain PY79 (Youngman et al., 1984) genomic DNA as a template, primer pairs PfrIISAS/PfrIISAA and PfrIISBS/PfrIISBA were used for PCR amplification of a 572 bp long sequence upstream of *spoIISA* and a 624 bp long sequence downstream of *spoIISB*, respectively. Resulting PCR products were cloned into EcoRI/BamHI and PstI/HindIII sites of pUS19, respectively, creating the pUS19-FRIIS plasmid. A fragment encoding resistance to kanamycin was PCR-amplified from the pUK19 plasmid (Ju et al., 1998) using primers PKnS/PKnA and subsequently cloned into PstI/BamHI sites of pUS19-FRIIS to obtain the pUS19-ΔIIS plasmid. Plasmid pUS19-ΔIIS was then integrated by double homological recombination into the chromosome of *B. subtilis* PY79 strain as described previously (Harwood and Cutting, 1990), creating IB1235 strain.

DNA sequence coding for the N-terminal part of SpoIISA (N-IISA) was PCR-amplified using primers PNIISAS and PNIISAA from chromosomal DNA of PY79 and cloned into KpnI/EcoRI sites of pSG1154 plasmid downstream of the xylose-inducible promoter creating the pSG1154-NIISA plasmid. This plasmid was integrated by double homological recombination into the *amyE* locus of the *B. subtilis* MO1099 strain (Guérout-Fleury et al., 1996) creating the IB1237 strain.

Plasmid pACYC-IISAB was constructed by insertion of the 1761 bp long NarI–HindIII fragment from plasmid pDS34-IISAB (a gift from Patrick Stragier) into plasmid pACYC184 (Chang and Cohen, 1978).

### 2.3 Immunodetection

*B. subtilis* cultures were inoculated from a fresh overnight plate to an OD_600_ of 0.1, grown as liquid cultures in appropriate medium and subsequently pelleted for 20 min at 1500 × *g.* Cell membranes were isolated similarly as described by Saller et al. (2009): pellets were resuspended in solubilization buffer (20 mM Tris–Cl, pH 8.0, 100 mM NaCl, 1 mM EDTA, 1 mM AEBSF, 0.25 mg ml^−1^ lysozyme) and after 10 min incubation on ice, cells were disrupted by sonication and cleared by centrifugation for 5 min at 1500 × *g*. The resulting supernatant was further centrifuged for 2 h at 60 000 × *g*. After ultracentrifugation, pellets were solubilized in an appropriate volume of preserving buffer (50 mM Tris–Cl pH 8.0, 20% glycerol) and stored at −80 °C. The volume of preserving buffer was calculated according to the OD_600_ of the original cell culture to obtain the same final total concentration of proteins in all tested samples.

Samples were separated by polyacrylamide gel electrophoresis (PAGE) in denaturing 12.5% polyacrylamide gels (Laemmli, 1970) and the proteins were transferred onto HyBond-ECL membranes (Amersham Pharmacia Biotech). Membranes were probed either using mouse polyclonal antibody raised against the cytosolic part of SpoIISA (dilution 1:500) to detect intact SpoIISA protein or using monoclonal anti-GFP antibody (Roche; dilution 1:1000). Detection of the proteins was carried out using horseradish peroxidase conjugated to goat anti-mouse secondary antibody (1:5000; Promega) with the ECL kit (Amersham Biosciences).

### 2.4 Fluorescence microscopy

GFP fluorescence in *B. subtilis* cells was investigated using an Olympus BX61 microscope equipped with an Olympus DP30BW camera and Olympus cell^P^ imaging software. For image deconvolution and figure rendering, the Huygens Professional software package was used.

### 2.5 Plasmid stability test

The effect of the *B. subtilis spoIIS* locus on plasmid stability was studied in serial plating experiments similarly to those performed previously in *Enterococcus faecium* and *Bacillus thuringiensis*, respectively (Grady and Hayes, 2003). During these experiments, single colonies of freshly transformed *E. coli* cells harboring either the parental pACYC184 plasmid or the pACYC-IISAB plasmid were streaked onto LB plates prepared with and without chloramphenicol, respectively. For this experiment, 8 clones from each transformation were assessed. After overnight incubation, one colony from each transformant subculture outgrown on non-selective LB plate was again streaked onto both non-selective and selective LB plates. The procedure was repeated 7 times with colonies outgrown on non-selective LB plates streaked onto both selective and non-selective plates. The subculture was defined as an overnight formation of a single colony corresponding to about 25 generations. The number of these discontinuous subcultures outgrown on selective medium was normalized to the number of subcultures outgrown on non-selective medium and was calculated in percentage. This experiment was repeated three times.

## 3 Results and discussion

### 3.1 Analysis of SpoIISA accumulation during the life cycle of *B. subtilis*

**Fig. 1:**
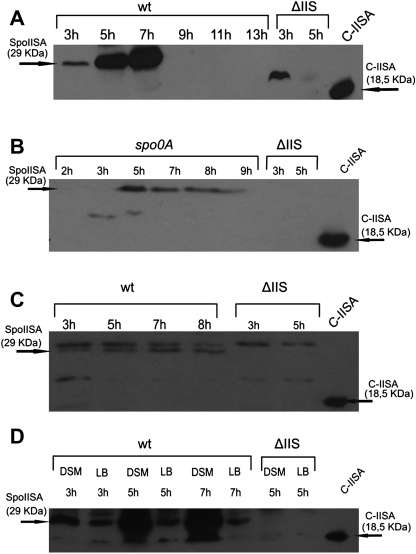
(A) Western blot analysis of SpoIISA in the membrane fraction of *B. subtilis* cells cultivated in DSM. The first six lanes represent SpoIISA levels at the indicated times (in h) after inoculation into the wild type PY79 strain. (B) Western blot analysis of SpoIISA in the membrane fraction of *B. subtilis* cells cultivated in DSM. The first six lanes represent the 2nd to the 9th h of growth of the IB220 strain (*spo0A* null mutant). (C) Western blot analysis of SpoIISA presence in the membrane fraction of *B. subtilis* cells grown in LB medium. Lanes 1 to 4 represent wild-type cells harvested 3–8 h after inoculation of the culture. (D) Comparison of SpoIISA presence in the wild type PY79 strain cultivated in DSM and LB medium, respectively. ΔIIS designates the IB1235 strain (*spoIIS* null mutant); C-IISA designates the lanes with 5 ng of a purified C-terminal part of SpoIISA. All signals other than those marked by arrows are non-specific.

SpoIISA toxin was first identified as a protein which, when produced in the absence of SpoIISB, is able to block sporulation at stage II. Despite the implied role in sporulation, one study showed that both *spoIIS* genes are transcribed during vegetative growth, with the highest expression levels being achieved approximately at the time of the onset of sporulation (Adler et al., 2001). A more recent transcriptional profiling analysis indicated that expression of the *spoIISA* gene might be under the control of σ^K^, the latest-acting of the sporulation sigma factors (Steil et al., 2005). To examine the possibility that SpoIISA could in some way be involved in sporulation, we monitored SpoIISA levels during different phases of the *B. subtilis* life cycle. According to the assumption that SpoIISA is a membrane protein (our further results showed that SpoIISA is indeed a membrane protein), we prepared membrane fractions of cells (see Material and methods) following growth in different media. SpoIISA was detected by western blot analysis using anti-SpoIISA antibodies. Initially, cells were grown in Difco sporulation medium (DSM) from which the onset of sporulation was assigned according to the growth curve. Samples were taken and analyzed at different times during growth and sporulation. As shown in Fig. 1A, the cells started to produce SpoIISA protein during the exponential phase of growth; however, a substantial increase in production of the toxin was observed approximately at the point of initiation of the sporulation process (5 h after inoculation). The peak of SpoIISA accumulation was detected during the 1^st^ 2 h of sporulation (7 h after inoculation); however we did not observe any signal corresponding to SpoIISA after the 4^th^ h of sporulation (9 h after inoculation). The absence of SpoIISA could be explained by the fact that our method is not optimal for the detection of membrane proteins, particularly in the later stages of sporulation, despite whether total cells or only membrane fractions were used for analysis. Subsequently we sought to determine whether SpoIISA expression was dependent on initiation of sporulation. For this, we used a *B. subtilis* strain with a deletion in *spo0A* which is unable to sporulate (Ireton et al., 1993) and in which sporulation is blocked at stage 0, prior to asymmetric division (Levin and Losick, 1996). Fig. 1B demonstrates that SpoIISA is present in the IB220 strain (*spo0A* null mutant), grown in DSM, from the beginning of stationary phase (the 5^th^ h of growth) to the 9^th^ h of growth when the cells begin to lyse. Since the overall amount of SpoIISA is higher in the wild type (Fig. 1A) than in the *spo0A* mutant (Fig. 1B), there is a possibility that Spo0A plays some role in SpoIISA expression. On the other hand, the explanation for these observations could be that SpoIISA turnover is faster in Spo0A-depleted cells than in the wild-type cells and therefore we see the stronger signal for SpoIISA in the latter.

Our results concerning the timing of SpoIISA production (Fig. 1A) are consistent with previous observations of Adler et al. (2001), although we still cannot exclude the possibility that SpoIISA plays some role during the later stages of sporulation. It is quite conceivable that SpoIISA may accelerate mother cell putrefaction and thus facilitate the release of the mature spore into the environment, at the same time rendering scarce nutrients for use by kin cells, so as to complete their own sporulation process or to sustain growth of non-sporulating cells.

**Fig. 2:**
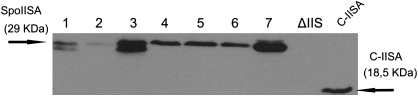
Effect of various salts added to LB medium on expression of SpoIISA toxin. Cells in all lanes were harvested from LB and DSM media, respectively after 5 h of growth. Lane 1 represents cells grown in LB medium; lane 2, LB medium supplemented with 0.1% KCl and 0.012% MgSO_4_; lane 3, LB medium supplemented with 1 mM Ca(NO_3_)_2_, 10 μM MnCl_2_ and 1 μM FeSO_4_; lane 4, LB medium supplemented with 0.1% KCl, 0.012% MgSO_4_, 1 mM Ca(NO_3_)_2_, 10 μM MnCl_2_ and 1 μM FeSO_4_; lane 5, LB medium supplemented with 10 μM MnCl_2_ and 1 mM Ca(NO_3_)_2_; lane 6, LB medium supplemented with 10 μM MnCl_2_; lane 7 represents cells grown in DSM medium. ΔIIS designates IB1235 strain (*spoIIS* null mutant) grown in LB medium and harvested after 5 h of growth. C-IISA designates a lane with 5 ng of purified cytosolic part of SpoIISA.

We next examined how SpoIISA is produced in the non-sporulation medium, LB, where we expected the same profile of SpoIISA accumulation as in DSM medium. However, as shown in Fig. 1C, we observed a very weak SpoIISA signal during all 8 h of growth in LB medium. When we compared the signal intensities from cells grown in LB and DSM, respectively, we observed a similar amount of SpoIISA in both media only at the 3^rd^ h of growth (Fig. 1D). Thereafter, there was an approximately one order of magnitude increase in the signal after 5 h growth for cells in DSM. It appears that the cellular SpoIISA concentration somehow depends on the composition of the media. We therefore tested the influence of various combinations of the five salts present in DSM on expression of SpoIISA in cells grown in LB (Fig. 2). We obtained a very weak SpoIISA signal when KCl and MgSO_4_ were added (Fig. 2 lane 2), weaker than in LB alone (Fig. 2 lane 1). On the other hand, when 1 mM Ca(NO_3_)_2_, 10 μM MnCl_2_ and 1 μM FeSO_4_ were added (Fig. 2 lane 3), the SpoIISA signal was almost as strong as in DSM (Fig. 2, lane 7). The other combination of salts added to LB (Fig. 2, lanes 4 and 5) increased the signal compared to LB without any salts, but did not overcome the signal when only MnCl_2_ was added to the medium (Fig. 2, lane 6). Taken together, these results indicate that KCl and MgSO_4_ decrease the level of SpoIISA while, on the other hand, Ca(NO_3_)_2_, MnCl_2_ and FeSO_4_ increase the amount of SpoIISA in the cells. Currently, we cannot predict the mechanism by which expression of the SpoIISA toxin is influenced by MnCl_2_, Ca(NO_3_)_2_ and FeSO_4_ salts.

### 3.2 Analysis of SpoIISB during the life cycle of *B. subtilis* cells

SpoIISB production during the life cycle of wild type *B. subtilis* cells in DSM media was monitored. Under these conditions, we were unable to detect SpoIISB using a standard western blot method, which was used for analysis of SpoIISA toxin. There exists the possibility that SpoIISB is rapidly turned over; however, it was shown previously that SpoIISB can be detected with a SpoIISB antibody when the protein is overexpressed in both *B. subtilis* (unpublished data) and *E. coli* (Florek et al., 2008).

### 3.3 Localization of SpoIISA

SpoIISA is predicted to be associated through its three putative transmembrane domains with the cytoplasmic membrane (Adler et al., 2001). We investigated this prediction using western blot analysis and fluorescence microscopy.

**Fig. 3:**
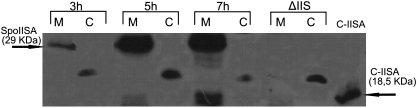
Analysis of the presence of SpoIISA in the membrane (M) and cytosolic fractions (C) of *B. subtilis* cells grown in DSM medium. The first six lanes designate PY79 wild-type cells at different h of growth where initiation of sporulation corresponds to 5 h of growth. The seventh and the eighth lanes designate the IB1235 strain (*spoIIS* null mutant) harvested after 5 h of growth. C-IISA designates a lane with 5 ng of purified cytosolic part of SpoIISA.

Immunoblotting analysis of cell membrane and cytosolic fractions revealed the presence of SpoIISA signals exclusively in *B. subtilis* membrane fractions (Fig. 3; lanes 1, 3, 5), establishing that SpoIISA is anchored in the cell membrane.

To confirm this finding, we sought to determine the in vivo localization of SpoIISA C-terminally fused to GFP using fluorescence microscopy. The observed green fluorescence signal was distributed throughout the cells and subsequent western blot analysis of total cell fractions showed that this signal corresponded to free GFP and not to the SpoIISA-GFP function protein (data not shown). One possible explanation for this observation is that GFP mRNA was transcribed from the P*_B_* promoter, which is responsible for expression of the *spoIISB* gene. To prevent expression of GFP from the P*_B_* promoter, we prepared a fusion of the N-terminal region of SpoIISA (N-IISA), including the transmembrane segments, with GFP. This construct was cloned into vector pUS19 and the localization of the fusion protein (N-IISA-GFP) was investigated. Even though the presence of N-IISA-GFP was detected at the beginning of stationary phase on immunoblots (data not shown), fluorescent microscopy revealed only a background signal in cells. Thus we conclude that probably only low amounts of N-IISA-GFP are produced under these conditions from the P*_AB_* promoter.

**Fig. 4:**
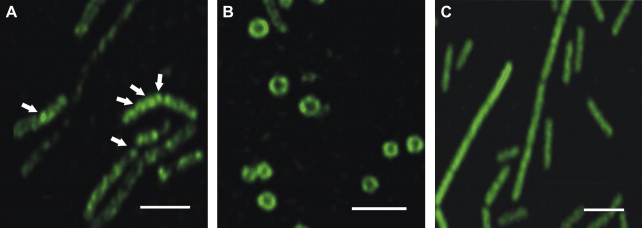
Localization of the N-terminal part of SpoIISA in *B. subtilis*. (A) Strain IB1237 expressing N-IISA in fusion with GFP. Foci of the fluorescence signal in the cytoplasmic membrane are depicted with arrows. (B) Protoplasts prepared from strain IB1237. (C) Strain IB1073 expressing GFP alone. Expression of N-IISA-GFP and GFP, respectively, was induced by 0.5% xylose. Scale bars represent 4 μm.

To increase the fluorescence signal, we cloned DNA encoding the first 86 amino acid residues corresponding to the transmembrane region of SpoIISA downstream from a xylose-inducible promoter into vector pSG1154 forming strain IB1237 (see Materials and methods). This time, when the fusion protein was overexpressed, we observed quite strong fluorescence signals which appeared to be associated with the cell membrane and forming distinct foci (Fig. 4A). Subsequently, we investigated strain IB1073, in which GFP alone was placed under control of the P*_xyl_* promoter (Table 1). In this instance, the GFP signal was exclusively associated with the cytoplasm without foci formation (Fig. 4C) These results, together with immunoblotting using anti-GFP antibody, proved that the fusion protein does not undergo significant degradation (data not shown) and is present in the cell membrane fractions. This observation confirms that SpoIISA is anchored in the cell membrane via its transmembrane parts. We next prepared protoplasts of *B. subtilis* cells expressing the N-IISA-GFP fusion protein. We observed fluorescent signals throughout the cell membrane with no foci apparent (Fig. 4B). The determinants of foci are not known, though they may be inherent in SpoIISA. However, the absence of foci in protoplasts suggests that their formation and/or stabilization could be influenced either by peptidoglycan or another component present in the cell wall.

### 3.4 The spoIIS locus does not act as an addiction system in *E. coli*

SpoIIS systems are present on chromosomes of many Bacillus species; however, their biological role is not clear. To further investigate the function of the SpoIIS system, we tested its possible involvement in plasmid stabilization by postsegregation killing (PSK). This phenomenon requires that the toxin component has a longer cellular half-life than the antitoxin. In these instances, bacteria become addicted to continuous expression of the antitoxin and consequently there is plasmid retention by the cells. PSK could not be observed in vegetatively growing *B. subtilis* cells, as they are immune to the activity of SpoIISA; therefore, we tested possibility that in *E. coli*, the presence of the *spoIIS* locus might increase the stability of the plasmid that harbors it. If the SpoIISAB module functions as a PSK system, it would be expected that the rate of loss of the plasmid bearing the TA module would be lower than the rate of loss of the parental plasmid. On the other hand, if cells shed the plasmid bearing the TA locus faster than or at the same rate as they shed the parental plasmid, this would suggest that the toxin is less stable than the antitoxin or that the stability of both proteins is similar.

**Fig. 5:**
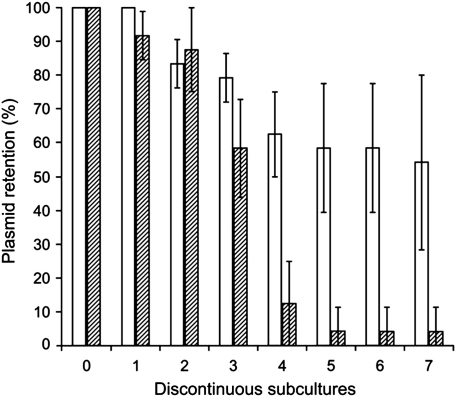
Effect of *B. subtilis spoIIS* on plasmid stability in *E. coli*. Strains carrying a plasmid without (white bars) and with *spoIIS* locus (hatched bars) were tested as described in Materials and methods. The number of subcultures resistant to chloramphenicol was normalized to the number of subcultures outgrown on plates without antibiotic and was calculated in percentages. The data presented are the means of three independent experiments and standard deviations are shown as error bars.

As shown in Fig. 5, the number of cells retaining plasmid pACYC-IISAB declines in the population considerably faster than those retaining the empty parental plasmid (pACYC184). These results indicate that the SpoIIS system does not function as an addiction module through PSK in *E. coli*. However, it was observed previously that the SpoIISA protein is degraded in *E. coli* by the activity of some proteases (Florek et al., 2008), which can prevent functioning of the SpoIIS system as an addiction module in this microorganism.

## Figures and Tables

**Table 1:** Bacterial strains and plasmids used in this study.

Strains/plasmids	Description	Source/reference
Strains		
*E. coli*		
MM294	*F-*, *endA-1*, *hsdR-17*, *(rk-*,*mk)*, *supE44*, *thi-1*,*recA+*	Backman et al. (1976)
IB 805	MM294 transformed with pACYC184	This study
IB 1058	MM294 transformed with pACYC-IISAB	This study

*B. subtilis*		
PY79	Prototrophic derivative of *B. subtilis* 168	Youngman et al. (1984)
MO1099	*amy*::*erm*	Guérout-Fleury et al. (1996)
IB220	*spo0A*::*kan*	Schmeisser et al. (2000)
IB1073	MO1099 *amy*::*P_xyl_-gfp-spc*	Pavlendová et al. (2010)
IB1235	PY79 *spoIIS*::*kan*	This study
IB1237	MO1099 *amy*::*P_xyl_-NIISA-gfp-spc*	This study

Plasmids		
pUS19	pUC19 with Sp^R^	Benson and Haldenwang (1993)
pUS19-FRIIS	pUS19 with flanking regions upstream of *spoIISA* and downstream of *spoIISB*	This study
pUS19-ΔIIS	pUS19-FRIIS with *kan*	This study
pSG1154	*bla amyE3′spc P_xyl_-gfp amyE5′*	Lewis and Marston (1999)
pSG1154-NIISA	*bla amyE3′spc P_xyl_-*sequence of 86 N-terminal residues of SpoIISA-*gfp amyE5′*	This study
pACYC184	Cm^R^ Tc^R^	Chang and Cohen, 1978
pACYC-IISAB	Cm^R^ Tc^R^*spoIIS*	This study

**Table 2:** PCR primers used in this study (restriction sites are underlined).

Name	Oligonucletides (5′ → 3′)	Restriction site/mutation
PfrIISAS	GATGATGATGAATTCAGACAGC AACTTGGCGAA	EcoRI
PfrIISAA	GATGATGATGGATCCACCGCTCC TTTGACAGAA	BamHI
PfrIISBS	GATGATGTACTGCAGACAAGGGAAAA CAGCTC	PstI
PfrIISBA	GATGATGTAAAGCTTCTGCAAGAGTGG AACAA	HindIII
PKnS	GATGATGATCTGCAGCCGCATCAGGCGATAAAC	PstI
PKnA	GATGATGATGGATCCGAATGGCGAATGCGCATAC	BamHI
PNIISAS	GATGATGATGGTACCGTTTTATTCTTTCAGATCATGGTC	KpnI
PNIISAA	GATGATGATGAATTCGACATAT GCACTTAAGAAGATAA	EcoRI
